# Solving The Problem of Fogged Eyewear in Orthopedic Surgeries in the COVID Scenario

**DOI:** 10.30476/BEAT.2021.86869.1171

**Published:** 2021-01

**Authors:** Rizwan Khan, Arvind Kumar, Mukesh Kumar, Javed Jameel

**Affiliations:** 1 Department of Orthopaedics, Hamdard Institute of Medical Sciences and Research, New Delhi, India

**Dear Editor,**

The current COVID-19 situation has been taking a high toll on the healthcare workers who are at constant risk of exposure to the contagion, especially those who are involved in treating the COVID-19 infected patients. The risk of exposure is further high during the surgical procedures, considering the aerosol generation and prolonged contact with the patients. Performing orthopedic surgeries in PPEs (personal-protective-equipment) is not an easy task. Besides the cumbersome body coverage of PPE gown and hood, and the N95 masks which result in early fatigue and exhaustion, a constantly frustrating issue is the fogging of eyewear which limits surgeon’s vision and ability to perform fine tasks [1]. In the current scenario, changing or removing eyewears, even temporarily, would mean adding the risk of contamination of eyes and indirectly of nasal pathway as well. A brief discussion regarding the problem of fogging of eyewear during surgical procedures and ways to prevent the same on the basis of currently available literature and our experience has been provided.

1. Why does fogging occur and why will it be more in COVID era?

Orthopedic surgeries often involve physical exertion and consequently, the skin perspiration increases, which creates a moisture around the skin. More heat results in increased activity of sweat glands and results in higher perspiration. The cooler surfaces near to skin with a relatively lower temperature (e.g, protective eyewear) tend to condense the moisture resulting in fogging. Additionally, the mask fit doesn’t guarantee a foolproof seal and the expired air may leak from the nasal bridge side when the contour of the mask fails to fit with that of the nose [2]. The expired air may leak into the eyewear, further adding to the moisture. With higher filtering capacity masks being used in the COVID era, both inspiration and expiration need extra efforts and carry a higher risk of air leakage if they are not properly sealed. Lastly, the currently recommended temperature of operation-theatre between 24°C and 30°C and the relative humidity between 40%-70%, both contribute to increased skin perspiration and fogging during surgery[3].

2. Why do conventional techniques for fogging prevention may not be useful in the current scenario? What are the possible solutions?

Few techniques have been described to prevent fogging of the eyewear. The basic principle described is to improve the seal of the mask that prevents the expired air from the mask to enter into eyewear [4]. This would have been effective in the pre-COVID era considering the predominantly non-hermetic nature of eyewear which allowed ventilation around the eyewear and prevented fogging due to skin perspiration. In the current scenario, the masks are hermetically sealed with no exchange between inner air to the outer air. Moreover, the above-discussed conditions of the operating room promote the fogging of the eyewear. Nevertheless, there are potential techniques which preserve the hermetic nature of seals while allowing some exchange of the filtered air. These techniques can potentially reduce the fogging of the eyewear. Vents/holes can be created at appropriate locations that do not hinder the surgical view and similar sized cut-outs from an N95 mask can be taped to the edges of the vents/holes from inside and outside. This will allow filtered air to circulate through those vents and the fabric part will absorb the moisture of the inner air, thus reducing the fogging [5]. Further, novel designs of filtered eye wears have been suggested but they are yet to be tested in real surgical situations.

3. Can anti-fog material help in preventing fogging?

The use of anti-fog coated eyewear has been frequently recommended [1]. Titanium dioxide coating has been widely used as an antifogging measure for the eyewear because of its hydrophilic nature and self-cleaning ability due to photocatalysis [6]. However, with the complete absence of Ultraviolet rays, this property may be lost. Unfortunately, the simulation studies have found that the majority of anti-fogging coated eyewear fails to prevent fogging even for a few seconds [7]. Therefore, further research is needed to develop eyewear coatings that can withstand long surgeries without fogging of their surface.

The vents of the goggles sealed with n95 cutting can be helpful in the current scenario with limited resources. Perspiration from skin increases with exertion and therefore, a wider clearance from skin may be helpful. Further research and development in this regard is needed. Considering the prolonged use of goggles in orthopedic surgeries, the selection should be based on ease of the fit as well. Special attention should be given to the softness of the sealing area as that remains in the contact with the skin throughout the surgery. Lastly, considering the heterogeneous and limited evidence of the effectiveness of the safety year in prevention of fogging, the surgeon should try the goggles in a simulated operating room environment to avoid intraoperative hassle.

## Conflict of Interest:

None declared.

## References

[1] Crebolder JM, Sloan RB (2004). Determining the effects of eyewear fogging on visual task performance. Appl Ergon..

[2] Lam SC, Lui AK, Lee LY, Lee JK, Wong KF, Lee CN (2016). Evaluation of the user seal check on gross leakage detection of 3 different designs of N95 filtering facepiece respirators. Am J Infect Control..

[3] Malhotra N, Bajwa SJS, Joshi M, Mehdiratta L, Trikha A (2020). COVID Operation Theatre- Advisory and Position Statement of Indian Society of Anaesthesiologists (ISA National). Indian J Anaesth..

[4] Jordan DJ, Pritchard-Jones R (2014). Tying a surgical mask to prevent fogging. Ann R Coll Surg Engl..

[5] Douglas D, Douglas R (2020). Addressing the corona virus pandemic: will a novel filtered eye mask help?. Int J Infect Dis..

[6] Duran IR, Laroche G (2019). Current trends, challenges, and perspectives of anti-fogging technology: Surface and material design, fabrication strategies, and beyond. Progress in Materials Science..

[7] Dain SJ, Hoskin AK, Winder C, Dingsdag DP (1999). Assessment of fogging resistance of anti-fog personal eye protection. Ophthalmic Physiol Opt..

